# Acute carotid stent thrombosis

**DOI:** 10.15171/jcvtr.2018.42

**Published:** 2018-12-05

**Authors:** Muzaffer Kahyaoglu, Murat Velioglu, Cetin Gecmen, Arzu Kalayci, Ender Ozgun Cakmak, İbrahim Akin Izgi

**Affiliations:** ^1^Department of Cardiology, Kartal Kosuyolu Heart & Research Hospital, Istanbul, Turkey; ^2^Department of Radiology, Kartal Kosuyolu Heart & Research Hospital, Istanbul, Turkey

**Keywords:** Thrombosis, Carotid Stent, t-PA, Tirofiban

## Abstract

Carotid artery stenting is a method used in the treatment of extracranial carotid artery stenosis that is becoming increasingly more common. Acute carotid thrombosis following CAS is a very rare and devastating complication that can be lethal for the patient unless treated immediately. We report a case of acute carotid stent thrombosis occurring immediately after emergent revascularization, and that was treated with intraarterial tissue plasminogen activator and intravenous tirofiban infusion.

## Introduction

Carotid artery stenting (CAS) is a method used in the treatment of extracranial carotid artery stenosis that is becoming increasingly more common.^1^ As with every method, complications related to the procedure can arise, the most common being cerebral embolization. Acute carotid thrombosis following CAS is a very rare and devastating complication that can be lethal for the patient unless treated immediately.^2^ The prevalence of this complication varies between 0.04% and 2.00%.^3^ Herein, we report a case of acute carotid stent thrombosis occurring immediately after emergent revascularization, and that was treated with intraarterial tissue plasminogen activator (t-PA) and intravenous (iv.) tirofiban infusion.

## Case Report

**Figure 1:**
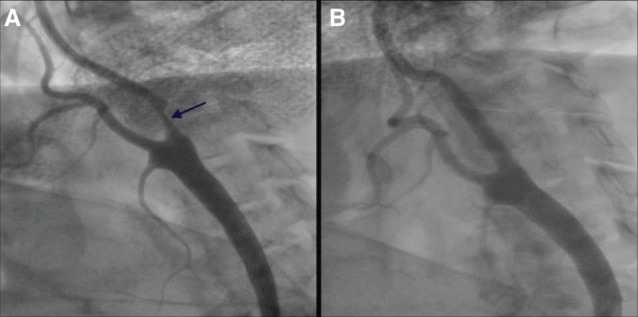
(A) Selective carotid angiography showing stenosis (arrow) of left internal carotid artery. (B) Closed cell self-expandable stent was implanted successfully.

**Figure 2:**
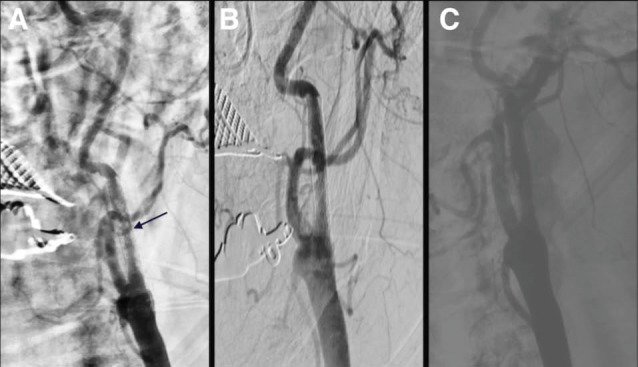
(A) Digital subtraction angiography showing stent thrombosis (arrow). (B) Stent thrombosis was partially resolved after medication. (C) Stent thrombosis was nearly resolved six hours later.

A 66-year-old male patient was admitted to our clinic presenting right hemiparesis and dysarthria. His medical history revealed that he had an ischemic stroke eight years previously. His complaints were fully diagnosed after 30 minutes. Cerebral computed tomography (CT) revealed chronic infarction in the right hemisphere. Cranial diffusion magnetic resonance imaging showed acute ischemic focus in the left hemisphere. As a result of these findings, the transient ischemic attack was diagnosed and he underwent selective carotid angiography. Angiography demonstrated 70% focal stenosis of the left internal carotid artery (LICA) (Figure 1A). He then underwent angioplasty of the LICA stenosis. Acetylsalicylic acid (100 mg/d) and clopidogrel (75 mg/d) were administered for seven days before the procedure. A total of 75 U/kg of unfractionated heparin was administered during the procedure and the Activated Clotting Time value was measured as 275 seconds. A distal protection device (EPI Embolic Protection Inc., Boston Scientific Corporation) was inserted using the transfemoral approach. A 6 to 8 × 40 mm closed cell self-expandable stent (Abbott Vascular, Santa Clara, CA) was implanted and post-dilated using a 5.0 × 20 mm balloon (Figure 1B). Three hours later, the patient developed motor aphasia and right hemiplegia. An emergent cerebral CT scan ordered did not reveal any signs of intracerebral hemorrhage. However, we learned from his deepened anamnesis that he had not taken acetylsalicylic acid and clopidogrel from the start, because he had not adhered to his medical therapy. The patient was then urgently transferred to the catheter laboratory where digital subtraction angiography (DSA) and selective carotid angiography revealed acute carotid stent thrombosis (Figure 2A). After the patient was given 300 mg of clopidogrel, 75 U/kg of unfractionated heparin was administered intravenously and selectively set into the carotid using the transfemoral approach. t-PA of 7 mg was slowly pushed into the internal carotid artery using the intraarterial selective approach and partial lysis was observed in the thrombus on DSA imaging performed after medication (Figure 2B). The patient was taken to the coronary intensive care unit and put on tirofiban infusion. Six hours later, subsequent DSA was performed and showed nearly complete recanalization of the LICA (Figure 2C). Cerebral CT ordered after seven days showed a small infarction. He was discharged with left arm weakness on the tenth post-procedure day with clopidogrel 75 mg 1x1 and acetylsalicylic acid 100 mg.

## Discussion

We describe a case in which the stenting of the extracranial ICA was complicated by acute thrombosis. Carotid stent thrombosis is an uncommon but very severe source of profound morbidity and mortality.^4^ Acute stent thrombosis remains a major concern for carotid stent implantation, occurring at a rate of 0.04% to 2.00%.^3^ Its etiology is multifactorial and has not been fully identified to date. Possible causes include drug interaction, drug compliance, antiplatelet resistance, genetic polymorphism, local vessel dissection, severe plaque protrusion, vasospasm, intimal injury, technical intraprocedural parameters, individual biological, and inflammatory factors.^4^

There are many reports of acute carotis stent thrombosis due to clopidogrel resistance. The response to clopidogrel varies widely among patients. Lack of responsiveness to clopidogrel has been reported to be in the range 4%–44%, depending on the test used and the limit values. In another study, clopidogrel-resistance rates ranged from 37% to 52% in patients who were undergoing cerebrovascular stent placement. Clopidogrel resistance is a frequent finding in the clinical context of neurovascular stent placement and seems to be associated with an increased risk of thromboembolic complication. However, in our case, we think that acute thrombosis of the carotid artery was due to inadequate antiaggregant therapy rather than clopidogrel resistance or technical procedural issues in our patient, on whom we performed the procedure without complication.

There are several previous reports about treatment modalities for carotid stent thrombosis. These are: intraarterial thrombolysis, surgical management, and endovascular treatment.^5-7^ Peri-interventional carotid stent thrombosis requires rapid intraarterial–intracarotid initiation of reperfusion therapy to limit the time of ischemia, reperfusion injury, and associated symptomatic intracranial hemorrhage.^8^ In our cases, acute carotid stent thrombosis was treated with intraarterial t-PA and intravenous tirofiban infusion. Each targets different phases of thrombus formation, making concurrent use relevant to revascularization. t-PA therapy targets the later stages of the coagulation cascade in disrupting the fibrin and thrombin network required for the formation of a hemostatic plug. In doing so, transient thrombin and platelet activation can cause a hypercoagulable phase that results in reocclusion.^9^ GP IIb/IIIa inhibitors to block expressed GP IIb/IIIa receptors on activated platelets, thereby preventing the important cross-bridging required for platelet aggregation, and inhibiting early vessel reocclusion.^10,11^

In conclusion, after t-PA and iv. tirofiban infusion, our patient was discharged with a minimal neurological disability. Acute carotid stent thorombosis is a rare but devastating complication of CAS, and rapid invasive diagnosis and reperfusion ensure good neurological recovery.

## Competing interests

The authors have no conflict of interest.

## Ethical approval

Patient data was anonymized and informed consent of the patient was taken.
